# Facial Preparation of Cyclometalated Iridium (III) Nanowires as Highly Efficient Electrochemiluminescence Luminophores for Biosensing

**DOI:** 10.3390/bios13040459

**Published:** 2023-04-04

**Authors:** Yueyue Huang, Egan H. Doeven, Lifen Chen, Yuanyuan Yao, Yueliang Wang, Bingyong Lin, Yanbo Zeng, Lei Li, Zhaosheng Qian, Longhua Guo

**Affiliations:** 1 College of Chemistry and Material Sciences, Zhejiang Normal University, Jinhua 321004, China; 2 Jiaxing Key Laboratory of Molecular Recognition and Sensing, College of Biological, Chemical Sciences and Engineering, Jiaxing University, Jiaxing 314001, China; 3 School of Life and Environmental Sciences, Faculty of Science, Engineering and Built Environment, Deakin University, Waurn Ponds, VIC 3216, Australia

**Keywords:** electrochemiluminescence, iridium(III) nanowire, biosensing, *Salmonella enteritidis*

## Abstract

In this study, highly efficient ECL luminophores composed of iridium complex-based nanowires (Ir–NCDs) were synthesized via covalently linking bis(2-phenylpyridine)-(4-carboxypropyl-2,2′-bipyridyl) iridium(III) hexafluorophosphate with nitrogen-doped carbon quantum dots (NCDs). The ECL intensity of the nanowires showed a five-fold increase in ECL intensity compared with the iridium complex monomer under the same experimental conditions. A label-free ECL biosensing platform based on Ir–NCDs was established for *Salmonella enteritidis* (SE) detection. The ECL signal was quenched linearly in the range of 10^2^–10^8^ CFU/mL for SE with a detection limit of 10^2^ CFU/mL. Moreover, the relative standard deviations (RSD) of the stability within and between batches were 0.98% and 3.9%, respectively. In addition, the proposed sensor showed high sensitivity, selectivity and stability towards SE in sheep feces samples with satisfactory results. In summary, the excellent ECL efficiency of Ir–NCDs demonstrates the prospects for Ir(III) complexes in bioanalytical applications.

## 1. Introduction

Electrochemiluminescence (ECL) is the luminescent emission resulting from highly exergonic electron transfer reactions between electrochemically generated oxidised and reduced species in the vicinity of the electrode surface [1]. ECL has some significant advantages; for example, no external light source is required, so the optical background noise is extremely low, and the sensitivity is very high. ECL has become a very powerful detection technique for a wide range of bio-related species via the ability to use ECL luminophores as labels in antibody/antigen or nucleic acid assays [2,3]. As a result, the technique has been widely used in the areas of clinical diagnostics, and life science research [4]. Recently, iridium(III) complexes have been investigated as alternative ECL reagents to the commonly employed ruthenium(II) complexes, due to various advantageous properties such as high photoluminescence efficiency, easily tuned emission colour, and large stokes shifts [5,6]. It was reported that (pq)_2_Ir(acac) showed greater ECL intensity than Ru(bpy)_3_^2+^ with tri-n-propylamine (TPA) co-reactant under optimum conditions (pq = 2-phenylquinoline anion, acac = acetylacetonate anion) [7]. However, their application in bio-assays has been limited due to their poor aqueous solubility and the limited availability of derivatives with bio-conjugatable ligands. The in-depth studies of cyclometalated iridium (III) complexes and their application in bioassays are still a challenge.

To address these challenges, the research focus has been devoted to synthesizing cyclometalated iridium(III) complex-based materials with higher ECL efficiency and water solubility, which is often accomplished by functionalizing their ancillary ligands using hydrophilic groups or by doping the iridium complex into a nanomaterial. For example, Li et al. have reported that [(ppy)Ir(dcbpy)^+^]PF_6_ can be applied for detection of cancer cells due to its water-solubility, enabled by the addition of a carboxyl substituted ancillary ligand [8]. Kerr et al., has synthesized water soluble Ir(III) complexes via functionalizing ligands with methylsulfonate or tetraethylene glycol groups [9]. Li et al., reported that water-soluble iridium(III) diimine complexes with appended sugar moieties can be used as efficient ECL luminophores for detection of antibiotics [10]. These characteristics help to improve the detection sensitivity of the ECL method. However, the synthetic process is complicated and time-consuming. Therefore, Liang et al. synthesized water-soluble nanoparticle by encapsulating Ir(III) complexes into organosilica nanoparticles [11]; however, the low emission intensity of the embedded Ir(III) complexes limited their further application. Employing apoferritin as a host, a water-soluble iridium complex-based bio-probe with strong ECL emission was designed by Yang’s group through encapsulating fac-tris(2-phenylpyridine)iridium(III) complexes [Ir(ppy)_3_] in the apoferritin (apoFt) cavity [12]. Chen’s group prepared water-soluble Ir nanorods (Ir NRs) via carboxyl-functionalization of tris (2-phenylpyridine) iridium(III) (Ir(ppy)_3_); the Ir nanorods were applied as anodic emitters for detecting organophosphorus pesticides [13]. However, despite these advances, the development of Ir(III) complexes for biosensing in the ECL field remains in its infancy.

Salmonellosis enteritidis, caused by *Salmonella enterica* (SE), is a food-borne pathogen-induced disease that poses a significant public health burden. The gut of various domestic and wild animals is the natural host for this pathogen [14,15]. Statistically, 70~80% of bacterial food poisoning in domestic situations is caused by salmonella [16,17]. Once animal products containing a large amount of salmonella (10^5^~10^6^ CFU/g) are digested by people, bacterial infection often results, and then food poisoning occurs under the action of toxins, leading to gastroenteritis, typhoid and paratyphoid [18,19]. Meat, eggs and other foods are all highly susceptible to contamination throughout the food supply chain from processing to sale. SE is considered as an important cause of bacterial intestinal disease in humans and livestock [20,21,22]. Therefore, achieving sensitive and accurate detection of SE is of high importance. Various methods including enzyme-linked immunosorbent assays [23,24], electrochemistry [25], fluorescence [26,27] and electrochemiluminescence [28,29] assays have been explored for detection of SE. Especially the ECL technique has been considered as a superior choice because of its remarkable sensitivity and controllability. To date, there have been no reports regarding Ir(III) complex-based ECL strategies for SE detection.

**Scheme 1:**
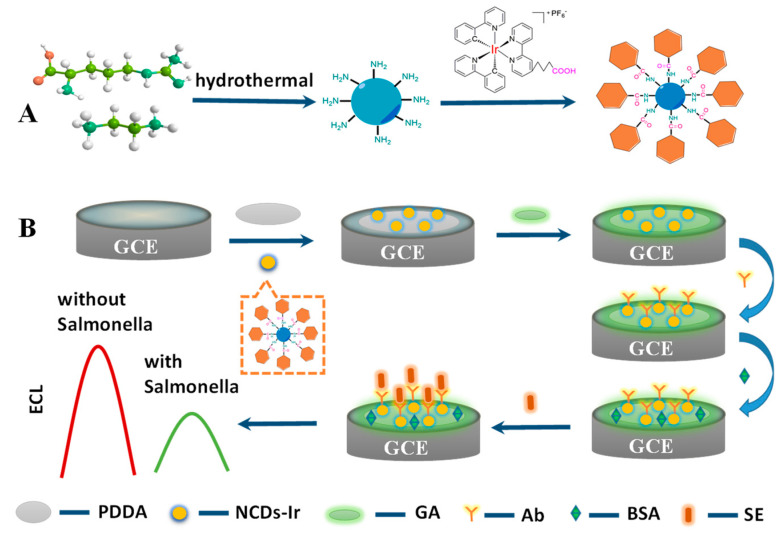
(**A**) Preparation ECL luminophore Ir–NCDs nanowire. (**B**) Fabrication process of biosensor.

Here, this work firstly prepared water-soluble Ir nanowires with a high luminous efficiency via a covalent linkage between a carboxyl-functionalized iridium complex and NCD, denoted as Ir–NCDs. As shown in Scheme 1A, firstly, nitrogen-doped carbon quantum dots were synthesized by a hydrothermal method using L-arginine and ethylenediamine as raw materials. Nitrogen-doped carbon quantum dots have plentiful amine groups, which enable facile amidation with the carboxyl bearing iridium complex, synthesizing Ir–NCDs nanowires. The Ir–NCDs exhibited superior ECL emission relative to the iridium complex monomer because of the enhanced contribution from NCDs to the ECL of the iridium complex. A novel label-free ECL platform was constructed with Ir–NCDs as the ECL luminophore, with SE selected as the analytical target. As shown in Scheme 1B, the Ir–NCDs nanowires are immobilized on the glassy carbon electrode (GCE), exhibiting efficient ECL. Poly(diallyldimethylammonium chloride) (PDDA) was subsequently dropped on the electrode surface to stabilize the film-form of Ir–NCDs and maintain signal stability, followed by glutaraldehyde (GA), allowing facile attachment of the SE antibody to the film surface. In the presence of SE, the antibody specifically recognizes the antigen and forms an antigen–antibody complex, insulating the GCE surface and hindering the electron transport process, thereby quenching the ECL signal. This label-free sensing strategy enabled the simple detection of SE with excellent selectivity and high stability.

## 2. Materials and Methods

### 2.1. Materials

Bis(2-phenylpyridine-C2,N)(4-carboxypropyl-2,2′-bipyridyl)iridium(III) hexafluorophosphate was synthesized and purified by SunaTech Inc, Suzhou, China. *Vibrio alginolyticus* (VA), *Vibrio parahaemolyticus* (VP), *Escherichia coli* (*E*. *coli*), *Vibrio sinaro* (VS) and *Salmonella enteritidis* (SE) were provided by the College of Animal Science and Technology, Guangxi University. *Salmonella enteritidis* antibody was purchased from Changzhou Zhongxin Biotechnology Co., Ltd., Hebei, China. Ethylenediamine and L-arginine were bought from Sinopharm Co., Ltd., Shanghai, China. 1-(3-DimethyLaminopropyl)-3-ethylcarbodiimide hydrochloride (EDC), glutaraldehyde (GA), polydiallyl dimethyl ammonium chloride (PDDA), bovine serum albumin (BSA) and N-hydroxysuccinimide (NHS) were purchased from Sigma-Aldrich, Saint Louis, MO, USA. The water used throughout was purified using a Millipore system, Burlington, MA, USA (resistivity 18 MΩ·cm).

### 2.2. Instruments

Transmission electron microscopy, used to image the morphologies of the synthesized nanomaterials, was performed using a Talos F200X (Thermo Fisher Scientific, Waltham, MA, USA) microscope. Ultraviolet-visible spectrometry was performed using a UV-vis spectrometer (UV-2700, Shimadzu, Kyoto, Japan). A fluorescence spectrophotometer (Shanghai Cold Light Technology, Shanghai, China, F97pro) was used to obtain the fluorescence spectrum of raw materials and products. Scanning electron microscopy, used to take topography and mapping of materials, was performed using a Hitachi S-4800 SEM (Hitachi, Tokyo, Japan). The constructed sensor was tested by electrochemical impedance spectroscopy (EIS) and cyclic voltammetry (CV) using an electrochemical workstation (Xian Remai). ECL measurements were performed on an MPI-E analyzer (Xi’an Ruimai Analytical Instruments Co., Ltd., Xi’an, China) using a traditional three-electrode system. In the three-electrode system, a glassy carbon electrode (GCE) was used for the working electrode. The sensor was constructed with a platinum wire counter electrode, and Ag/AgCl formed the reference electrode (saturated KCl). Solutions were adjusted to the correct pH with a 920 Precision pH meter.

### 2.3. Synthesis of NCDs and Ir–NCDs Nanowires

The amine-rich carbon dots (NCDs) were synthesized via the hydrothermal method according to previous work [30]. Briefly, the L-arginine and ethylenediamine were successively dissolved in ultrapure water and mixed. This mixed solution was then poured into a sealed Teflon lined reaction chamber, and the solution was then heated for 12 h at the programmed temperature of 180 °C. During the heating process, the color of the solution changes from clear to yellow due to the formation of NCDs. The solution was then filtered using a filter membrane, and the product was further purified by dialysis (membrane cutoff Mw = 2 kDa) to remove the raw material and other products. The NCDs product was dried by freeze-drying and stored in a refrigerator.

Ir–NCDs products are obtained by amidation of the carboxyl group of the iridium complex with the amine group of NCDs. The iridium complex is added to the synthetic NCDs solution, and the solution is mixed via ultrasonication until it becomes clear and transparent. EDC/NHS was added to activate the carboxyl group, and then stirred at room temperature for 16 h to obtain an orange-red product. The product is called Ir–NCDs. As a control experiment, Ir complex and NCDs are simply mixed without the addition of EDC/NHS, negating the formation of the Ir–NCDs. This Ir/NCDs mixture is used to compare ECL signals with Ir–NCDs and confirm the ECL enhancement from the nanowire formation. The mass concentration of iridium atoms in the Ir–NCDs was determined by an inductively coupled plasma emission spectrometer (ICP), and the molar concentration of the iridium complex was calculated by a linear regression equation (Figure S1 in Supplementary Materials).

### 2.4. Fabrication of ECL Sensor

The glassy carbon electrode was polished in turn with 0.3 μM and 0.05 μM alumina slurries, and then rinsed sequentially with ultrapure water, ethanol and acetone followed by ultrasonication before use. Then GCE electrodes were activated and further cleaned in 0.5 mol/L H_2_SO_4_ solution by CV (scanning from 1.0~−1.0 V) until an ideal voltammogram was obtained (no further change in background current). To construct the sensor, Ir–NCDs and PDDA (1:1) were mixed and sonicated for 30 min, and then 10 μL of the mixed solution was drop-coated on the glassy carbon electrode. PDDA is a polyelectrolyte and acts to increase the stability of the Ir–NCDs, while its conductive property enhances the electron transport to the modified electrode. The electrode was then drop-coated with 10 μL of GA, which acts as an intermediate linker between the Ir–NCDs and the antibodies. Subsequentially, 5 μL of antibody solution was drop-coated on the GCE surface and reacted overnight in a refrigerator at 4 °C. Finally, 10 μL of BSA was dropped on the electrode surface to react for 2 h, blocking the unbound sites. Before analysis, different concentrations of salmonella were incubated with the modified electrode for 1 h.

## 3. Results

### 3.1. Characterization of the NCDs and Ir–NCDs

**Figure 1:**
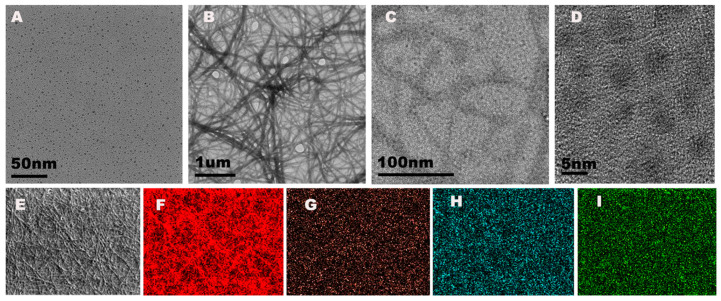
(**A**) TEM image of NCDs; (**B**) TEM image of Ir–NCDs; (**C**,**D**) magnified topography of Ir–NCDs at different magnifications; (**E**) original image of mapping characterization of Ir–NCDs; and (**F**–**I**) elemental distribution of Ir, C, N, and O, respectively.

The morphology of the NCDs and Ir–NCDs were characterized by TEM. The TEM image and the dark field image of the as-prepared NCDs showed spherical morphology with monodispersing properties (Figure 1A and Figure S2). Dynamic light scattering analysis showed that the average diameter ranged from 2 to 5 nm with an average diameter of 3.0 ± 0.4 nm (Figure S2). The high-resolution TEM image indicated a lattice spacing of 0.21 nm (Figure S2), which was consistent with the in-plane lattice spacing of graphene (100 facet). UV-visible absorption of NCDs showed two characteristic peaks centered at 280 nm and 358 nm, which were attributed to the π–π* transition of C=C and the n–π* transition f C=O (Figure S5).

The TEM image of the Ir–NCDs showed nanowires morphology due to the cross-linking between one iridium complex and multiple amine-rich Ir–NCDs (Figure 1B). The enlarged images of Figure 1C,D further demonstrate that the NCDs can be successfully encapsulated into the nanofibers. The EDS mapping analyses were carried out to analyze the surface elements of the nanowires (Figure 1E–I). As anticipated, the characteristic elements of Ir, C, N, and O were visible. The specific position and element distribution at spot 1 and spot 2 are shown in Figure S3. Additionally, the morphology of the iridium monomer in its solid state and solution was characterized by TEM (Figure S4). The iridium complexes formed a crystal structure in the solid state and were spherical in shape in solution, which further confirmed that the iridium complex can form nanowires due to the existent of NCDs. In addition, the UV-visible and fluorescence spectra were recorded to further confirm the successful synthesis of Ir–NCDs (Figure S5). The fluorescence spectrum of the Ir–NCDs showed two peaks at 430 and 580 nm, which was consistent with the fluorescence maximum emission wavelengths of NCDs and the Ir complex recorded separately.

### 3.2. ECL Performance of Ir–NCDs and Feasibility Study

**Figure 2:**
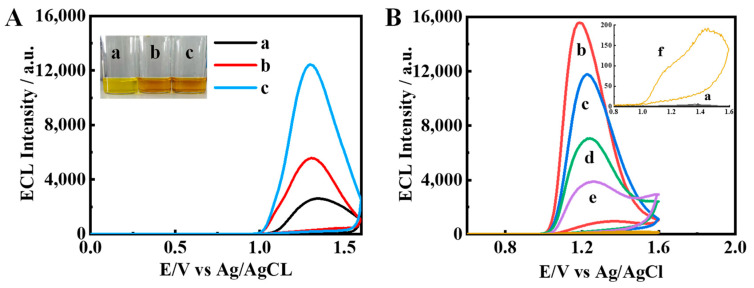
Comparison of ECL signals of (**A**) a: Ir complexes monomer (black), b: Ir/NCDs (red), and c: Ir–NCDs (blue); and (**B**) a: bare GCE; b: Ir–NCDs/PDDA; c: Ir–NCDs/PDDA/GA; d: Ir–NCDs/PDDA/GA/Ab; e: Ir–NCDs/PDDA/GA/Ab/BSA; f: Ir–NCDs/PDDA/GA/Ab/BSA/SE.

The ECL properties of the iridium monomer, Ir–NCDs (covalent bond) and Ir/NCDs (simple mixture) were compared. As shown in Figure 2A, the iridium complex alone exhibits weak ECL emission. After the addition of NCDs to the iridium complex solution, the ECL intensity of Ir/NCDs is nearly two times higher than that of the monomer, and the ECL intensity of Ir–NCDs is further increased to five times that of the iridium complex. These results suggest that the higher ECL efficiency of Ir–NCDs may be attributed to the contribution of the covalently bound NCDs. NCDs are a new type of quantum dots, which are environmentally friendly and contain a large number of amine groups that can be used as a co-reactant reagent with the iridium complex, thereby enhancing its ECL emission. After the amidation reaction, the electron transport distance between NCDs and the iridium complex was shortened, and the ECL intensity of the nanowires was improved significantly. The mechanism of ECL emission is shown in Figure S6.

The effect on the ECL of the stepwise construction of the sensor was studied by taking SE as an example. The results are shown in Figure 2B. First, the mixed solution of Ir–NCDs and PDDA was modified on the electrode to obtain relatively high ECL signal, and then glutaraldehyde (GA) was modified on the electrode, which resulted in a decrease in the ECL signal measured. Further modification of the modified layer with antibodies and BSA resulted in further reductions in the ECL signal due to the quenching effect of protein molecules. Finally, the specific binding of SE with the antibody further quenches the ECL, depending on the concentration of SE, indicating that the constructed ECL quenching sensor can realize the sensitive detection of SE.

### 3.3. Electrochemical Characterization of the Immunosensor

**Figure 3:**
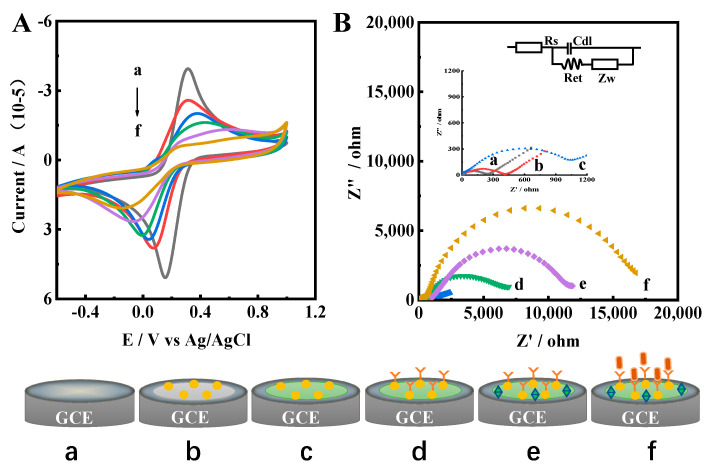
(**A**) CV and (**B**) EIS curves of a: bare GEC; b: Ir–NCDs/PDDA; c: Ir–NCDs/PDDA/GA; d: Ir–NCDs/PDDA/GA/Ab; e: Ir–NCDs/PDDA/GA/Ab/BSA; and f: Ir–NCDs/PDDA/GA/Ab/BSA/SE. The EIS spectra were obtained with a 5 mM [Fe(CN)_6_]^3−^/^4−^ solution using a frequency range of 0.1 MHZ–0.1 Hz, a bias potential of 0.225 V and an AC amplitude of 5 mV.

To investigate the assembly process of the immunosensor electrochemically, cyclic voltammetry was performed in 0.1 M PBS containing 5.0 mM K_3_[Fe(CN)_6_]/K_4_[Fe(CN)_6_] and 0.1 M KCl. As shown in Figure 3A, a decreased current response was found for Ir–NCDs/PDDA, indicating that the current was suppressed due to the electron-blocking effect of the Ir–NCDs/PDDA layer. After modification with GA, the current signal was further decreased, which is mainly due to the decreased electron transfer of the redox probe on the electrode surface. Weaker peak currents were observed after modifying with Ab. This is due to the successful assembly of the proteins on the electrode, which results in an additional electron-blocking layer of protein. After BSA modification on the electrode, the current value decreases further because BSA blocks the unreacted active sites. After the capture of SE at the electrode surface, a further subsequent decrease in current was observed due to insulting blocking of protein molecules by SE, which illustrates that the immunosensor for detection of SE was successfully constructed.

To further confirm the successful assembly of stepwise surface modification on the electrode surface (Figure 3B), electrochemical impedance spectroscopy (EIS) was performed in 0.1 M PBS containing 5.0 mM K_3_[Fe(CN)_6_]/K_4_[Fe(CN)_6_] and 0.1 M KCl using a frequency range of 0.1 MHz–0.1 Hz, an applied potential of 0.225 V, and an AC amplitude of 5 mV. According to previous reports [31], the equivalent circuit (as shown in the inset), was applied as of model to analyze the EIS spectra. The equivalent circuit included the electrolyte solution resistance Rs, the surface electron transfer resistance Ret, the Warburg impedance Zw and the double layer capacitance Cdl. The diameter of the semicircle of the Nyquist curve reflects the electron transfer resistance (Ret) at the electrode interface. Compared with the bare glassy carbon electrode (GCE, curve a), a small semicircle portion of the curve was observed for Ir–NCDs/PDDA. After modification with GA, the Ret increased due to the layer of GA. The assembly of Ab on the electrode results in a barrier to the interfacial charge, which further increases Ret. Further stepwise increases in the semicircle portion of the curve were observed after modification with BSA, likely originating from the partial insulation of the electrode by the BSA protein. After the incubation of the electrode with SE and subsequent binding of SE to Ab, the Ret is further increased, due to the relatively large particle size of *Salmonella enteritidis*. Thus, the transmission of electrons is easily hindered, and the current value also decreases significantly. The EIS spectrum was consistent with the CV results, which demonstrates the successful immobilization of each modification step.

### 3.4. Optimization of Experimental Conditions

**Figure 4:**
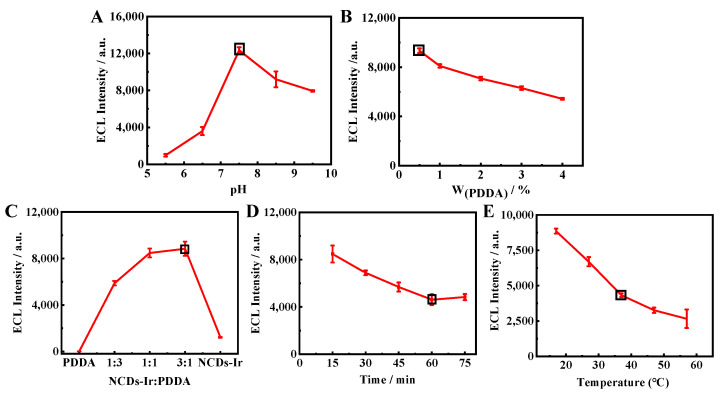
Optimization of the reaction conditions: (**A**) the pH of the PBS buffer solution; (**B**) the amount of PDDA; (**C**) the mixing ratio of Ir–NCDs and PDDA; (**D**) the incubation time of the modified antigen on the electrode; (**E**) the incubation temperature of the modified antigen on the electrode.

To obtain the best performance of the immunosensor, the pH of PBS, scan rate, amount of PDDA and ratio of the nanowires and PDDA, reaction time, and temperature were optimized. The corresponding results are shown in Figure 4. Firstly, the pH of PBS (0.10 M) was optimized. An increasing ECL signal from the Ir–NCDs nanowires was observed with the pH increasing from 5.0–7.5. When the pH surpassed 7.5, the ECL signal dropped gradually. Therefore, pH 7.5 was chosen as the optimal pH.

The effect of PDDA with different mass fractions (0.5%, 1%, 2%, 3%, 4%) on the ECL signals was studied. As shown in Figure 4B, the ECL signals decreased obviously with the increasing PDDA mass fraction. Therefore, 0.5% was chosen as the optimal mass fraction. As shown in Figure 4C, the ratio of PDDA and Ir–NCDs was also optimized. The responses of ECL signals increased with the ratio of PDDA and Ir–NCDs from 1:3 to 3:1, At the ratio of 1:1 for PDDA and nanowires, the immunosensor achieved the maximum ECL intensity. Therefore, Ir–NCDs:PDDA = 1:1 was determined as the optimum ratio.

As shown in Figure 4D, the ECL signal decreased significantly with increasing SE binding time. These results indicate that more SE was specifically bound to Ab as the incubation time increased. When the reaction time reached 60 min, the ECL signal remained stable, indicating that the reaction was basically saturated at 60 min. Therefore, 60 min was selected as the best incubation time. In addition, it can be seen from Figure 4E that the ECL signal also changes with the incubation temperature. Considering the activity of the antigen at a certain temperature, the incubation temperature was finally selected to be 37 °C.

### 3.5. Performance of Proposed Biosensor

**Figure 5:**
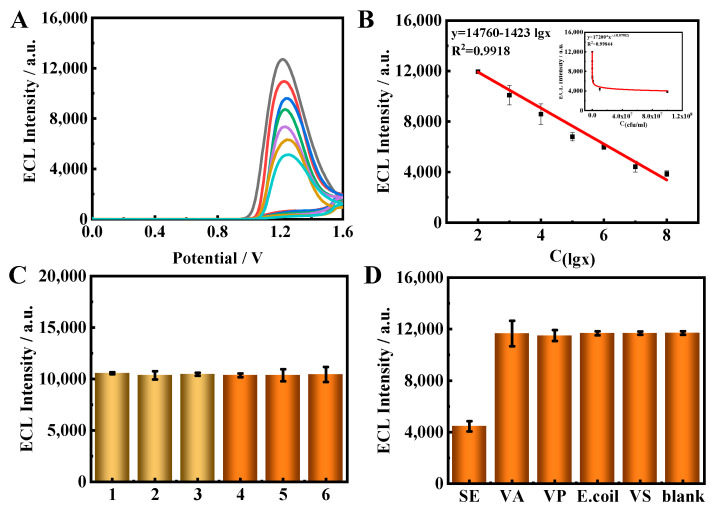
(**A**) ECL–time curves of the immunosensor towards different concentrations of targets: 1 × 10^2^, 1 × 10^3^, 1 × 10^4^, 1 × 10^5^, 1 × 10^6^, 1 × 10^7^ and 1 × 10^8^ CFU/mL. (**B**) Calibration curve of the sensor response to SE concentrations. (**C**) The stability of the proposed immune sensor to the target. (**D**) Selectivity of the proposed immunosensor for the target.

Based on the optimum conditions, the analytical performance of the immunosensor for detecting SE with various concentrations was investigated. As shown in Figure 5A, with the increase in SE concentration, the ECL from the Ir–NCDs at the electrode surface decreased, resulting in a quenching of the ECL signal. Figure 5B shows the linear relationship between the concentration of SE and the ECL intensity. The linear fitting equation was y = 14,760–1423 lgC, with a detection limit of 1 × 10^2^ CFU/mL (S/N = 3). Additionally, the LOD and linear range of detecting SE in this work was compared with various methods reported in the literature (Table S1), confirming that the proposed method showed higher sensitivity than other methods, with an excellent detection range.

The reproducibility of the immunosensor was evaluated via the intra-and inter-assays (Figure 5C). After the three modified electrodes were incubated with the same concentration of target, the relative standard deviations for the intra-batches and inter-batches were 0.98% and 3.9% respectively. Additionally, the ECL intensity was scanned six times using repetitive cyclic voltammetry. The relative standard deviation (RSD) was 1.68%. These investigations show the excellent stability and reproducibility of the proposed immunosensor.

The selectivity of the immunosensor for SE detection was demonstrated using five representative bacteria with similar structures, using *Vibrio alginolyticus* (VA), *Vibrio parahaemolyticus* (VP), *Escherichia coli* (*E*. *coli*) and *Vibrio sinairobi* (VS) as interferences. The concentrations were all 1 × 10^9^ CFU/mL, 100-fold larger than the SE sample. As shown in Figure 5D, The ECL signals of the interferences were found to be indistinguishable from that of the blank; however, the ECL intensity for SE showed a large decrease, highlighting the selectivity of this sensing approach. The results illustrate that the ECL signal was clearly decreased only in the presence of the target SE, showing the excellent specificity of the immunosensor.

### 3.6. Application of ECL Bacterial Sensor in Real Samples

To verify that the prepared bacterial sensor has good reliability in practical application, the SE concentration in sheep feces was quantified by the proposed immunosensor. The recovery test was performed by a standard addition method. A 1 g sample of sheep excrement was ultrasonically dispersed into 5 mL of 0.01 M PBS, followed by centrifugation. The supernatant was then collected and filtered. SE was spiked into the sheep feces sample to final concentrations of 1 × 10^8^, 1 × 10^7^, and 1 × 10^6^ CFU/mL before analysis. As shown in Table 1, the recovery rate was between 94.99% and 110.0%, indicating that the sensor yields accurate results from a complex sample matrix, demonstrating the promising practicality for determination of bacterial concentrations in real samples.

## 4. Conclusions

In this work, Ir–NCDs nanowires were synthesized through a simple cross-linking between amidation of the carboxyl groups of cyclometallic iridium complexes with the amino groups of nitrogen-doped carbon quantum dots. A reliable, sensitive and selective label-free ECL immunosensor based on the Ir–NCDs was proposed for detecting *Salmonella enteritidis* for the first time. Carbon dots provided an enhanced contribution to the ECL performance of Ir–NCDs due to efficient intramolecular electron transfer processes. The fabricated ECL immunosensor was constructed through a specific assembly between antigens and antibodies. The immunosensor has sufficient stability and selectivity for determination of SE with a detection limit as low as 10^2^ CFU/mL. Therefore, the immunosensor is expected to provide a new method for preparing a novel ECL luminophore and expanding the application of iridium complexes in bio-related analysis.

## Figures and Tables

**Table 1:** Recovery results of spiked salmonella in sheep feces sample.

Added (CFU/mL)	Found (CFU/mL)	Recovered (%)	RSD (%)
	1.100 × 10^5^	110.0%	
1 × 10^5^	1.012 × 10^5^	101.2%	6.214%
	0.9756 × 10^5^	97.56%	
	1.040 × 10^6^	104.0%	
1 × 10^6^	1.005 x10^6^	100.5%	2.403%
	0.9932 × 10^6^	99.32%	
	0.9780 × 10^7^	97.80%	
1 × 10^7^	1.041 × 10^7^	104.1%	4.714%
	0.9499 × 10^7^	94.99%	

## Data Availability

Data available on request from the authors.

## Supplementary Materials

The following supporting information can be downloaded at: https://www.mdpi.com/article/10.3390/bios13040459/s1, Figure S1: Quantitative analysis of Ir–NCDs by ICP-Ms Measurement. Figure S2: (A) Dark field image of nitrogen-doped carbon quantum dot NCDs. (B) Lattice diagram of nitrogen-doped quantum dots. (C) Particle size distribution of nitrogen-doped quantum dots. Figure S3: Elemental Analysis of Ir–NCDs. Figure S4: (A) SEM of iridium complex monomer in solid state. (B) SEM of iridium complex monomer in the C_2_H_5_OH solution. Figure S5: (A) UV-vis spectra of a: NCDs; b: Ir(III); and c: Ir–NCDs. (B) Emission spectra of a: NCDs; b: Ir(III); and c: Ir–NCDs. Table S1: Detection of *Salmonella enteritidis* by Different Methods. References [32,33,34,35,36,37,38,39] are cited in Supplementary Materials.

## References

[1] Liu Z., Qi W., Xu G. (2015). Recent Advances in Electrochemiluminescence. Chem. Soc. Rev..

[2] Hu L., Xu G. (2010). Applications and Trends in Electrochemiluminescence. Chem. Soc. Rev..

[3] Bhaiyya M., Pattnaik P.K., Goel S. (2021). A Brief Review on Miniaturized Electrochemiluminescence Devices: From Fabrication to Applications. Curr. Opin. Electrochem..

[4] Lv W., Ye H., Yuan Z., Liu X., Chen X., Yang W. (2020). Recent Advances in Electrochemiluminescence-Based Simultaneous Detection of Multiple Targets. TrAC Trends Anal. Chem..

[5] Swanick K.N., Ladouceur S., Zysman-Colman E., Ding Z. (2012). Self-Enhanced Electrochemiluminescence of an Iridium (III) Complex: Mechanistic Insight. Angew. Chem. Int. Ed..

[6] Xu Z.H., Gao H., Zhang N., Zhao W., Cheng Y.X., Xu J.J., Chen H.Y. (2020). Ultrasensitive Nucleic Acid Assay Based on Cyclometalated Iridium (III) Complex with High Electrochemiluminescence Efficiency. Anal. Chem..

[7] Chen L., Doeven E.H., Wilson D.J., Kerr E., Hayne D.J., Hogan C.F., Yang W., Pham T.T., Francis P.S. (2017). Co-Reactant Electrogenerated Chemiluminescence of Iridium (III) Complexes Containing an Acetylacetonate Ligand. ChemElectroChem.

[8] Li C., Lin J., Guo Y., Zhang S. (2011). A Novel Electrochemiluminescent Reagent of Cyclometalated Iridium Complex-Based DNA Biosensor and its Application in Cancer Cell Detection. Chem. Commun..

[9] Newman B., Chen L., Henderson L.C., Doeven E.H., Francis P.S., Hayne D.J. (2020). Water-Soluble Iridium (III) Complexes Containing Tetraethylene-Glycol-Derivatized Bipyridine Ligands for Electrogenerated Chemiluminescence Detection. Front Chem..

[10] Li M.J., Jiao P., Lin M., He W., Chen G.N., Chen X. (2011). High Electrochemiluminescence of a New Water-Soluble Iridium (III) Complex for Determination of Antibiotics. Analyst.

[11] Zanarini S., Rampazzo E., Bonacchi S., Juris R., Marcaccio M., Montalti M., Paolucci F., Prodi L. (2009). Iridium Doped Silica–PEG Nanoparticles: Enabling Electrochemiluminescence of Neutral Complexes in Aqueous Media. J. Am. Chem. Soc..

[12] Yang L., Sun X., Wei D., Ju H., Du Y., Ma H., Wei Q. (2020). Aggregation-Induced Electrochemiluminescence Bioconjugates of Apoferritin-Encapsulated Iridium (III) Complexes for Biosensing Application. Anal. Chem..

[13] Yang G., He Y., Zhao J., Chen S., Yuan R. (2021). Ratiometric Electrochemiluminescence Biosensor Based on Ir Nanorods and Cds Quantum Dots for the Detection of Organophosphorus Pesticides. Sens. Actuators B Chem..

[14] Li S., He Y., Mann D.A., Deng X. (2021). Global Spread of *Salmonella enteritidis* via Centralized Sourcing and International Trade of Poultry Breeding Stocks. Nat. Commun..

[15] Ranjbar R., Dehkordi F.S., Heiat M. (2020). The Frequency of Resistance Genes in *Salmonella enteritidis* Strains Isolated from Cattle. Iran. J. Public Health.

[16] Duguid J.P., North R.A. (1991). Eggs and *Salmonella* Food-Poisoning: An Evaluation. J. Med. Microbiol..

[17] Nurjayadi M., Pertiwi Y., Islami N., Azizah N., Efrianti U., Saamia V., Wiranatha I., Nastassya L., El-Enshasye H.A. (2019). Detection of the *Salmonella* Typhi Bacteria in Contaminated Egg Using Real-Time PCR to Develop Rapid Detection of Food Poisoning bacteria. Biocatal. Agric. Biotechnol..

[18] Aljamali N.M. (2021). Review on Food Poisoning (Types, Causes, Symptoms, Diagnosis, Treatment). Glob. Acad. J. Pharm. Drug Res..

[19] Hamad R., Saleh A.A. (2019). Incidence of Some Food Poisoning Bacteria in Raw Meat Products with Molecular Detection of *Salmonella* in Al Beida City, Libya. Alex. J. Vet. Sci..

[20] Walker N., Li S., Strauss H., Pokharel S. (2021). *Salmonella typhimurium* DT 104 Response to Lytic Bacteriophage and Lactobionic Acid on Raw Chicken Breast. Food Microbiol..

[21] Mthembu T.P., Zishiri O.T., El Zowalaty M.E. (2021). Genomic Characterization of Antimicrobial Resistance in Food Chain and Livestock-Associated *Salmonella* Species. Animals.

[22] Parker E.M., Parker A.J., Short G., O’Connor A.M., Wittum T.E. (2022). *Salmonella* Detection in Commercially Prepared Livestock Feed and the Raw Ingredients and Equipment Used to Manufacture the Feed: A Systematic Review and Meta-Analysis. Prev. Vet. Med..

[23] Zeinhom M.M.A., Wang Y., Sheng L., Du D., Li L., Zhu M.J., Lin Y. (2018). Smart Phone Based Immunosensor Coupled with Nanoflower Signal Amplification for Rapid Detection of *Salmonella enteritidis* in Milk, Cheese and Water. Sens. Actuators B Chem..

[24] Gu K., Song Z., Zhou C., Ma P., Li C., Lu Q., Liao Z., Huang Z., Tang Y., Li H. (2022). Development of Nanobody-Horseradish Peroxidase-Based Sandwich ELISA to Detect *Salmonella enteritidis* in Milk And in vivo Colonization in Chicken. J. Nanobiotechnol..

[25] Hasan M.R., Pulingam T., Appaturi J.N., Zifruddin A.N., Teh S.J., Lim T.W., Ibrahim F., Leo B.F., Thong K.L. (2018). Carbon Nanotube-Based Aptasensor for Sensitive Electrochemical Detection of Whole-Cell *Salmonella*. Anal. Biochem..

[26] Wan J., Zheng L., Kong L., Lu Z., Tao Y., Feng Z., Lv F., Meng F., Bie X. (2021). Development of A Rapid Detection Method for Real-Time Fluorescent Quantitative PCR of *Salmonella* spp. and *Salmonella enteritidis* in Ready-to-Eat Fruits and Vegetables. LWT—Food Sci. Technol..

[27] Zhang P., Liu H., Ma S., Men S., Li Q., Yang X., Wang H., Zhang A. (2016). A Label-Free Ultrasensitive Fluorescence Detection of Viable *Salmonella enteritidis* Using Enzyme-Induced Cascade Two-Stage Toehold Strand-Displacement-Driven Assembly of G-quadruplex DNA. Biosens. Bioelectron..

[28] Zhang R., Liang Y., Su Y., Lai W., Zhang C. (2021). Cloth-based Closed Bipolar Electrochemiluminescence DNA Sensors (Ccbedss): A New Class of Electrochemiluminescence Gene Sensors. J. Lumin..

[29] D’souza D., Jaykus L.A. (2003). Nucleic Acid Sequence Based Amplification for the Rapid and Sensitive Detection of *Salmonella enterica* from Foods. J. Appl. Microbiol..

[30] Filippini G., Amato F., Rosso C., Ragazzon G., Vega-Peñaloza A., Companyó X., Dell’Amico L., Bonchio M., Prato M. (2020). Mapping the Surface Groups of Amine-Rich Carbon Dots Enables Covalent Catalysis in Aqueous Media. Chem.

[31] Lu Y., Guo Z., Song J., Huang Q., Zhu S., Huang X., Wei Y. (2016). Tunable Nanogap Devices for Ultra-Sensitive Electrochemical Impedance Biosensing. Anal. Chim. Acta.

[32] Gao S., He L. (2019). Development of a filtration-based SERS mapping platform for specific screening of *Salmonella enterica* serovar Enteritidis. Anal. Bioanal. Chem..

[33] Kim T.H., Hwang H.J., Kim J.H. (2017). Development of a Novel, Rapid Multiplex Polymerase Chain Reaction Assay for the Detection and Differentiation of *Salmonella enterica* Serovars Enteritidis and Typhimurium Using Ultra-Fast Convection Polymerase Chain Reaction. Foodborne Pathog. Dis..

[34] Li F., Li F., Chen B., Zhou B., Yu P., Yu S., Lai W., Xu H. (2020). Sextuplex PCR Combined with Immunomagnetic Separation and PMA Treatment for Rapid Detection and Specific Identification of Viable *Salmonella* spp., *Salmonella Enterica* Serovars Paratyphi B, *Salmonella Typhimurium* and *Salmonella enteritidis* in Raw Meat. Food Control.

[35] Ren J., Man Y., Li A., Liang G., Jin X., Pan L. (2020). Detection of *Salmonella enteritidis* and *Salmonella typhimurium* in Foods Using a Rapid, Multiplex Real-Time Recombinase Polymerase Amplification Assay. J. Food Saf..

[36] Zang C.L., Zhang M.D., Zhang Y., Li Y.S., Liu K., Xie N.N., Sun C.Y., Zhang X.G. (2021). Rapid Label-Free Detection of *Salmonella Enterica* with Biolayer Interferometry. J. Food Saf..

[37] Wang Y., Zhang A., Yang Y., Lei C., Cheng G., Zou W., Zeng J., Chen Y., Wang H. (2018). Sensitive and Rapid Detection of *Salmonella Enterica* Serovar Indiana by Crosspriming Amplification. J. Microbiol. Methods.

[38] Draz M., Lu X. (2016). Development of a Loop Mediated Isothermal Amplification (LAMP)—Surface Enhanced Raman spectroscopy (SERS) Assay for the Detection of *Salmonella Enterica* Serotype Enteritidis. Theranostics.

[39] Lavu P.S.R., Mondal B., Ramlal S., Murali H.S., Batra H.V. (2016). Selection and Characterization of Aptamers Using a Modified Whole Cell Bacterium SELEX for the Detection of *Salmonella Enterica* Serovar Typhimurium. ACS Comb. Sci..

