# Supplementary material for: Facial Preparation of Cyclometalated Iridium (III) Nanowires as Highly Efficient Electrochemiluminescence Luminophores for Biosensing
Source: Biosensors (Basel). 2023 Apr 4;13(4):459. doi: 10.3390/bios13040459 (PMC10136454; doi:10.3390/bios13040459)
Supplement: Supplementary file 1 [file biosensors-13-00459-s001.zip › biosensors-2238511-supplementary.pdf]

Facial Preparation of Cyclometalated Iridium (III) Nanowires as Highly Efficient Electrochemiluminescence Luminophores for Biosensing

Yueyue Huang [1,2], Egan H. Doeven [3], Lifen Chen [2,*], Yuanyuan Yao [2], Yueliang Wang [2], Bingyong Lin [2], Yanbo Zeng [2], Lei Li [2], Zhaosheng Qian [1,*] and Longhua Guo [2,*]

1 College of Chemistry and Life Sciences, Zhejiang Normal University, Jinhua 321004, China
2 Jiaxing Key Laboratory of Molecular Recognition and Sensing, College of Biological, Chemical Sciences and Engineering, Jiaxing University, Jiaxing 314001, China
3 School of Life and Environmental Sciences, Faculty of Science, Engineering and Built Environment, Deakin University, Waurn Ponds, VIC 3216, Australia
* Correspondence: chenlf@zjxu.edu.cn (L.C.); qianzhaosheng@zjnu.cn (Z.Q.); guolh@fzu.edu.cn (L.G.)

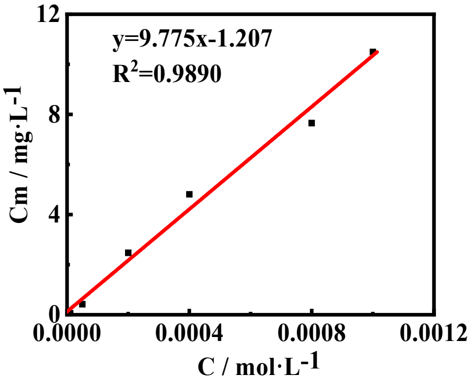

Figure S1. Quantitative analysis of Ir−NCDs by ICP-Ms Measurement.

In order to compare ECL signal intensity between Ir−NCDs nanowires and Ir complexes in the same concentration, it is necessary to determine the concentration of Ir complexes in Ir−NCDs nanowires. The x-coordinate is the molar concentration of the Ir complex, and the y-coordinate is the mass concentration of the Ir atom. The mass concentration of Ir atoms was measured by ICP−MS using a series of molar concentration of Ir complexes. Then, the mass concentration of Ir atoms of the synthesized Ir−NCDs nanowires was measured by ICP−MS, according to the linear diagram, the molar concentration of Ir complexes corresponding to the mass concentration of Ir atoms in Ir−NCDs nanowires was determined.

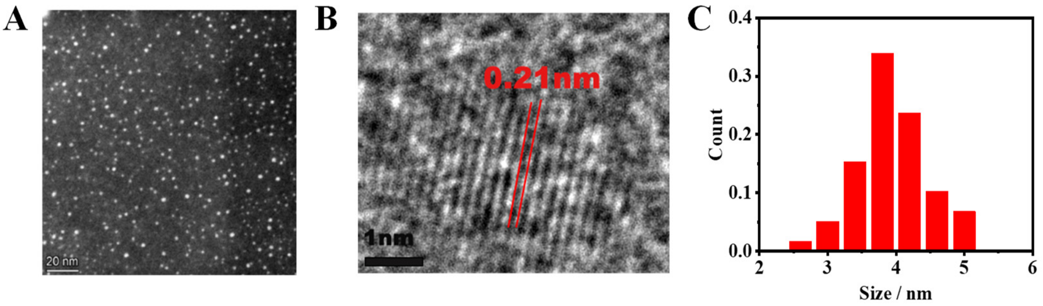

Figure S2. (A) Dark field image of nitrogen-doped carbon quantum dot NCDs. (B) Lattice diagram of nitrogen-doped quantum dots. (C) Particle size distribution of nitrogen-doped quantum dots.

A

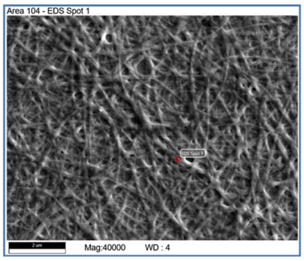

eZAF Quant Result - Analysis Uncertainty: 99.00 %

Element	Weight %	MDL	Atomic %	Error %	Net Int.	R	A	F
C K	58.9	0.16	80.7	6.4	1424.2	0.8934	0.7381	1.0000
N K	8.4	0.23	9.9	10.2	175.2	0.9026	0.4425	1.0000
O K	6. 9	0.15	7.1	8.6	237.0	0.9101	0.5893	1.0000
Ir M	25.7	0.62	2.2	6.1	208.3	0.9571	0.9761	1.0046

B

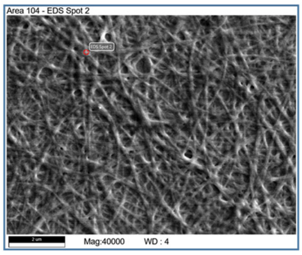

eZAF Quant Result - Analysis Uncertainty: 99.00 %

Element	Weight %	MDL	Atomic %	Error %	Net Int.	R	A	F
C K	60.0	0.15	81.3	6.8	1451.2	0.8885	0.6971	1.0000
N K	9.5	0.22	9.9	10.3	180.1	0.8979	0.4286	1.0000
O K	7.6	0.14	6.7	8.8	230.4	0.9056	0.5747	1.0000
Ir M	24.0	0.56	2.2	6.1	200.9	0.9545	0.9731	1.0046

Figure S3. Elemental Analysis of Ir−NCDs. (A) It's position and element distribution table at spot 1. (B) It's the position and element distribution table at spot 2.

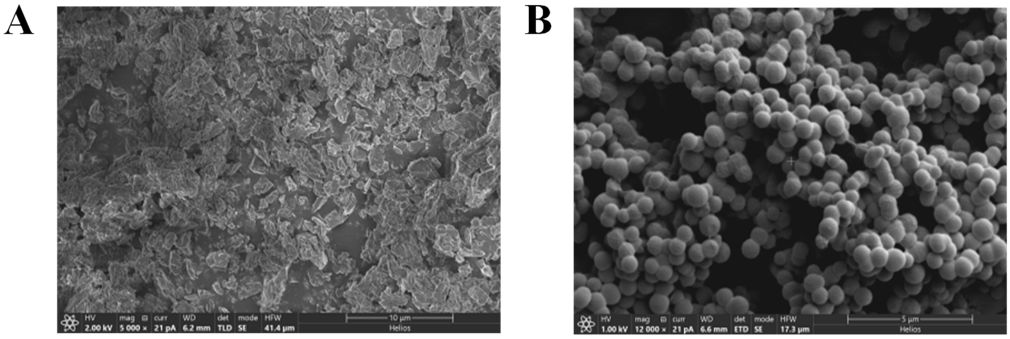

Figure S4. (A) SEM of iridium complex monomer in solid state. (B) SEM of iridium complex monomer in the C_2H_5OH solution.

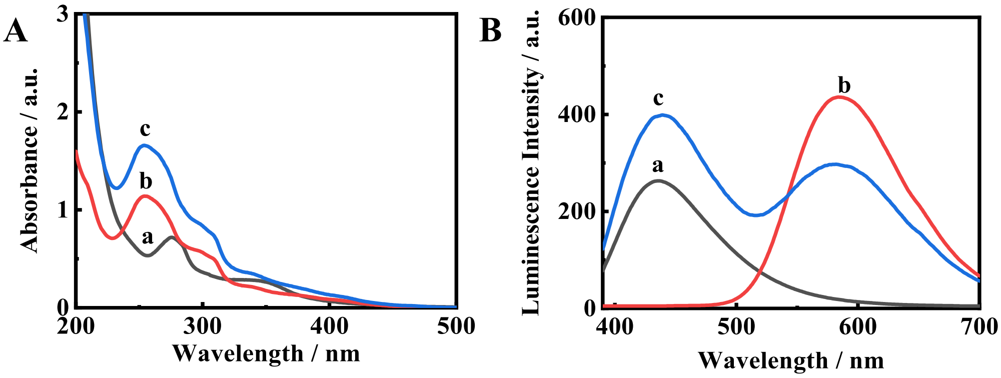

Figure S5. (A) Uv-vis spectra of a:NCDs; b: Ir(III); c: Ir−NCDs. (B) Emission spectra of a:NCDs; b: Ir(III); c: Ir−NCDs.

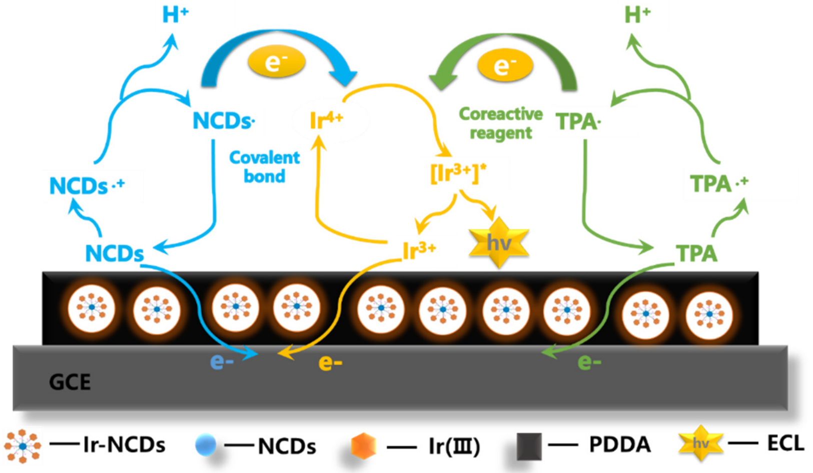

Figure S6. Electrochemical luminescence mechanism diagram of Ir complex.

The synthetic Ir–NCDs nanowires, which are prepared by covalently bonded between NCDs and Ir complexes, which can shorten the electron transport path to enhance ECL of Ir complexes. The reaction process is as follows:

$Ir^{3+} - e^{-} \rightarrow Ir^{4+}$ （S6-1）

$NCDs - e^{-} \rightarrow NCDs^{+\cdot}$ （S6-2）

$NCDs^{+\cdot}$ loses hydrogen ion H^{+}, and then becomes $NCDs^{\cdot}$ with high reducibility. The reaction process is as follows:

$NCDs^{+\cdot} - H^{+} \rightarrow NCDs^{\cdot}$ （S6-3）

Ir^{4+} and $NCDs^{\cdot}$ continue to react to obtain the excited state of Ir^{3+*} and NCDs, the reaction is as follows:

$Ir^{4+} + NCDs^{\cdot} \rightarrow Ir^{3+*} + NCDs$ （S6-4）

The excited Ir^{3+*} in the process of returning to the ground state Ir^{3+}, energy is released in the form of light as follows:

$Ir^{3+*} \rightarrow Ir^{3+} + h\nu$ （S6-5）

Tripropylamine TPA, as a co-reaction reagent, was dissolved in PBS solution to promote the ECL of Ir complex. When a certain voltage is applied to the outside world, the Ir complex on the electrode and the TPA in the solution lose electrons on the electrode. The reaction process is as follows:

$Ir^{3+} - e^{-} \rightarrow Ir^{4+}$ （S6-6）

$TPA - e^{-} \rightarrow TPA^{+\cdot}$ （S6-7）

$TPA^{+\cdot}$ loses hydrogen ion H^{+}, and then becomes $TPA^{\cdot}$ with high reducibility. The reaction process is as follows:

$TPA^{+\cdot} - H^{+} \rightarrow TPA^{\cdot}$ （S6-8）

Ir^{4+} and $TPA^{\cdot}$ continue to react, and the excited state of Ir^{3+*} and Product-1 is obtained. The reaction is as follows:

$Ir^{4+} + TPA^{\cdot} \rightarrow Ir^{3+*}$ + Products-1 （S6-9）

The excited Ir^{3+*} In the process of returning to the ground state Ir^{3+}, energy is released in the form of light as follows:

$Ir^{3+*} \rightarrow Ir^{3+} + h\nu$ （S6-10）

The methods of SE detection are summarized, and then the detection range and detection limits of various methods are compared. It can be seen that the sensor constructed in this paper can realize sensitive detection of SE.

Table S1 Detection of Salmonella Enteritidis by Different Methods

Targets	Detection method	Detection range	Limit of detection	Reference
SE	SERS	1.4×10^3-1.4×10^7 CFU/mL	10^3 CFU/mL	S1
SE	PCR	4.5-4.5×10^4 CFU/mL	4.5 CFU/mL	S2
SE	IMS-PMA-mPCR	1×10^1-1×10^7 CFU/mL	68 CFU/mL	S3
SE	RPA	1×10^2-1×10^6 CFU/mL	100 CFU/mL	S4
SE	biolayer interferometry	7.04×10^5-1.1×10^9 CFU/mL	1.17×10^4 CFU/mL	S5
SE	CPA-NADS	6.2-6.2×10^5 CFU/mL	62 CFU/mL	S6
SE	LAMP-SERS	6.6-6.6×10^6 CFU/mL	66 CFU/mL	S7
SE	SELEX	1×10^1-1×10^6 CFU/mL	100 CFU/mL	S8
SE	ECL	1×10^2-1×10^8 CFU/mL	100 CFU/mL	working

SERS:surface enhanced Raman spectroscopy; PCR:polymerase chain reaction; IMS:immunomagnetic separation; PMA:propidium monoazide; RPA:recombinase polymerase amplification; CPA:cross-priming amplifification; NADS:combined with a nucleic acid detection strip; NADS:nucleic acid detection strip; LAMP:Loop Mediated Isothermal Amplification; SELEX:systematic evolution of ligands by exponenttial enrichment;

Supporting references

S1. Gao. S, He. L, Development of a filtration-based SERS mapping platform for specific screening of Salmonella enterica serovar Enteritidis, *Anal. Bioanal. Chem*, **2019**, *411*, 7899–7906.

S2. T. H. Kim, H. J. Hwang, J. H, Development of a Novel, Rapid Multiplex Polymerase Chain Reaction Assay for the Detection and Differentiation of Salmonella enterica Serovars Enteritidis and Typhimurium Using Ultra-Fast Convection Polymerase Chain Reaction, *Foodborne Pathog Dis*, **2017**,*10*, 14.

S3. F. Li, F. Li, B. Chen, B. Zhou, P. Yu, S. Yu, W. Lai, H. Xu, Sextuplex PCR combined with immunomagnetic separation and PMA treatment for rapid detection and specific identification of viable Salmonella spp., Salmonella enterica serovars Paratyphi B, Salmonella Typhimurium and Salmonella Enteritidis in raw meat, *J food saf*, **2020**, *40*, 12784..

S4. J. Ren, Y. Man, A. Li, G. Liang, X. Jin, L. Pan, L, Detection of Salmonella enteritidis and Salmonella typhimurium in foods using a rapid, multiplex real-time recombinase polymerase amplification assay, *J food saf*, **2020**, *40*, 12784.

S5. C. L. Zang, M. D. Zhang, Y. Zhang, Y. S. Li, K. Liu, N. N. Xie, C. Y. Sun, X. G. Zhang, Rapid label-free detection of Salmonella enterica with biolayer interferometry, *J food saf*, **2021**, 41, e12896.

S6. Y. Wang, A. Zhang, Y. Yang, C. Lei, G. Cheng, W. Zou, J. Zeng, Y. Chen, H. Wang, Sensitive and rapid detection of Salmonella enterica serovar Indiana by crosspriming amplification, *J microbiol meth*, **2018**, *153*, 24–30.

S7. M. Draz, X. Lu, Development of a Loop Mediated Isothermal Amplification (LAMP) – Surface Enhanced Raman spectroscopy (SERS) Assay for the Detection of Salmonella Enterica Serotype Enteritidis, *Theranostics*, **2016**, *6 (4)*, 522-532.

S8. P. S. R. Lavu, B. Mondal, S. Ramlal, H. S. Murali, H. V. Batra, Selection and Characterization of Aptamers Using a Modified Whole Cell Bacterium SELEX for the Detection of Salmonella enterica Serovar Typhimurium, *ACS Comb*, **2016**, *18*, 292−301.
